# Ramadan intermittent fasting induced poorer training practices during the COVID-19 lockdown: A global cross-sectional study with 5529 athletes from 110 countries

**DOI:** 10.5114/biolsport.2022.117576

**Published:** 2022-06-27

**Authors:** Jad Adrian Washif, David B. Pyne, Øyvind Sandbakk, Khaled Trabelsi, Abdul Rashid Aziz, Christopher Martyn Beaven, Isabel Krug, Iñigo Mujika, Achraf Ammar, Anis Chaouachi, Imen Moussa-Chamari, Asma Aloui, Hamdi Chtourou, Abdulaziz Farooq, Monoem Haddad, Mohamed Romdhani, Paul Salamh, Montassar Tabben, Del P. Wong, Yacine Zerguini, Matthew D. DeLang, Lee Taylor, Helmi Ben Saad, Karim Chamari

**Affiliations:** 1 Sports Performance Division, Institut Sukan Negara Malaysia (National Sports Institute of Malaysia), 57000 Kuala Lumpur, Malaysia; 2 Research Institute for Sport and Exercise, University of Canberra, Canberra, Australia; 3 Centre for Elite Sports Research, Department of Neuromedicine and Movement Science, Norwegian, University of Science and Technology, Trondheim, Norway; 4 High Institute of Sport and Physical Education, University of Sfax, Sfax, Tunisia; 5 Research Laboratory: Education, Motricity, Sport and Health, EM2S, LR19JS01, University of Sfax, Sfax, Tunisia; 6 Sport Science and Sport Medicine, Singapore Sport Institute, Sport Singapore, Singapore; 7 Division of Health, Engineering, Computing and Science, Te Huataki Waiora School of Health, University of Waikato, Tauranga, New Zealand; 8 Melbourne School of Psychological Sciences, The University of Melbourne, Melbourne, Australia; 9 Department of Physiology, Faculty of Medicine and Nursing, University of the Basque Country, Leioa, Basque Country; 10 Exercise Science Laboratory, School of Kinesiology, Faculty of Medicine, Universidad Finis Terrae, Santiago, Chile; 11 Institute of Sport Sciences, Otto-von-Guericke University, 39104 Magdeburg, Germany; 12 Interdisciplinary Laboratory in Neurosciences, Physiology and Psychology: Physical Activity, Health and Learning (LINP2), UFR STAPS, UPL, Paris Nanterre University, Nanterre, France; 13 Tunisian Research Laboratory, Sport Performance Optimisation, National Center of Medicine and Science in Sports (CNMSS), Tunis, Tunisia; 14 AUT University, Sports Performance Research Institute New Zealand, Auckland, New Zealand; 15 Physical Education Department, College of Education, Qatar University, Doha, Qatar; 16 Physical Activity, Sport & Health Research Unit (UR18JS01), National Sport Observatory, Tunis, Tunisia; 17 High Institute of Sport and Physical Education, University of Gafsa, Tunisia; 18 Aspetar, Orthopaedic and Sports Medicine Hospital, FIFA Medical Centre of Excellence, Doha, Qatar; 19 Krannert School of Physical Therapy, University of Indianapolis, Indianapolis, USA; 20 School of Nursing and Health Studies, Hong Kong Metropolitan University, Hong Kong; 21 FIFA Medical Centre of Excellence Algiers, Algeria; 22 Medical Committee, Confederation of African Football, Egypt; 23 Right to Dream Academy, Old Akrade, Ghana Lee Taylor; 24 School of Sport, Exercise and Health Sciences, Loughborough University. National Centre for Sport and Exercise Medicine (NCSEM), Loughborough, United Kingdom; 25 Human Performance Research Centre, University of Technology Sydney, Sydney, Australia; 26 Sport & Exercise Discipline Group, Faculty of Health, University of Technology Sydney, Sydney, Australia; 27 Laboratoire de Recherche “insuffisance cardiaque” (LR12SP09), Hôpital Farhat HACHED, Université de Sousse, Sousse, Tunisie; 28 Laboratoire de Physiologie, Faculté de Médicine de Sousse, Université de Sousse, Sousse, Tunisie

**Keywords:** Crowdsource data, Global sports, Vulnerable athletes, Remote training, Training perception, Training load

## Abstract

Ramadan intermittent fasting during the COVID-19 lockdown (RIFL) may present unique demands. We investigated training practices (i.e., training load and training times) of athletes, using pre-defined survey criteria/questions, during the ‘first’ COVID-19 lockdown, comparing RIFL to lockdown-alone (LD) in Muslim athletes. Specifically, a within-subject, survey-based study saw athletes (n = 5,529; from 110 countries/territories) training practices (comparing RIFL to LD) explored by comparative variables of: sex; age; continent; athlete classification (e.g., world-class); sport classification (e.g., endurance); athlete status (e.g., professional); and level of training knowledge and beliefs/attitudes (ranked as: good/moderate/poor). During RIFL (compared to LD), athlete perceptions (ranges presented given variety of comparative variables) of their training load decreased (46–62%), were maintained (31–48%) or increased (2–13%). Decreases (≥ 5%, p < 0.05) affected more athletes aged 30–39 years than those 18–29 years (60 vs 55%); more national than international athletes (59 vs 51%); more team sports than precision sports (59 vs 46%); more North American than European athletes (62 vs 53%); more semi-professional than professional athletes (60 vs 54%); more athletes who rated their beliefs/attitudes ‘good’ compared to ‘poor’ and ‘moderate’ (61 vs 54 and 53%, respectively); and more athletes with ‘moderate’ than ‘poor’ knowledge (58 vs 53%). During RIFL, athletes had different strategies for training times, with 13–29% training twice a day (i.e., afternoon and night), 12–26% at night only, and 18–36% in the afternoon only, with ranges depending on the comparative variables. Training loads and activities were altered negatively during RIFL compared to LD. It would be prudent for decision-makers responsible for RIFL athletes to develop programs to support athletes during such challenges.

## INTRODUCTION

Healthy adult Muslims fast for 29–30 days each year during Ramadan [1]. Eating and drinking are not permitted between dawn (*imsak*) and sunset (*iftar*), a duration generally ~10–22 hours, dependent on geographical location [2, 3]. At extreme latitudes where an absence of sunrise/sunset occurs, clerical decree’s set fasting hours [4]. Ramadan intermittent fasting (RIF) through various religious and non-religious forms, particularly the former, modifies sleep-wake cycles [5] and eating patterns [6], generally disrupting ‘normal’ lifestyle [2] whilst compromising physical [1, 7] and cognitive performance [8]. Blood glucose levels, hydration status and availability of metabolites for short explosive and endurance physical efforts are likely sub-optimal [1, 6, 9] during this fasting period. These challenges are evidently more pronounced in athletic compared to sedentary populations undertaking RIF.

The coronavirus disease 2019 (COVID-19) pandemic altered everyday life for most of the globe [10, 11, 12]. Governmental countermeasures varied across the world [13]. Pertinent to athletes, movement restrictions or lockdowns occurred in many countries where the general population, including athletes, were encouraged (or obligated) to stay at home [10, 14]. Among wider populations, lockdowns affected quality of life, inducing depression [15], post-traumatic stress [16], and poor sleep quality [10, 14]. Athletes reported poorer sleep behaviours and decreased mental wellbeing during lockdown [17, 18, 19] alongside limited access to regular training, recovery, sports science and medical support, and potentially optimal nutrition [18, 20, 21]. Consequently, training practices among athletes (e.g., training intensity, frequency, and volume) were altered or compromised [19, 22]. Plausibly, RIF during the COVID-19-enforced lockdown (RIFL) may present greater challenges and/or effects on athlete training than lockdown-alone (LD).

Understanding changes in training practices related to RIFL is important, as it may inform evidence-based COVID-19 recommendations for future pandemics or lockdown-like situations, for athletes undertaking RIFL. Therefore, the influences of RIFL on training practices were assessed and compared to LD in athletes during the ‘first’ COVID-19 lockdown. Further, comparative variables were also explored, including: sex; age; continent; athlete classification (e.g., world class, national, state); sport classification (e.g., aquatic, combat, endurance, team); athlete status (e.g., amateur, semi-pro, professional); and level of training knowledge and beliefs/attitudes (ranked as: good, moderate, and poor). We hypothesised that RIFL would lower training loads compared to LD.

## MATERIALS AND METHODS

### Participants

**FIG. 1:**
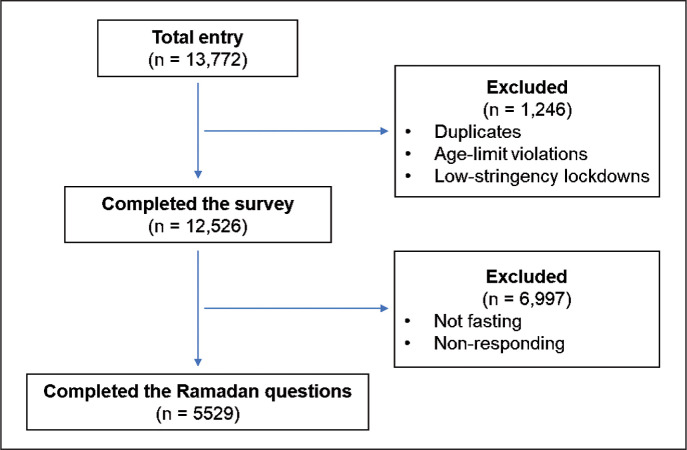
Flow chart of athlete’s recruitment.

A final sample of 5,529 athletes from 110 countries and territories, representing Muslim athletes that fasted during Ramadan in 2020 were included in the analysis (Figure 1). Participant eligibility criteria were: *(i)* Muslim athletes who fasted during Ramadan in April-May 2020; *(ii)* ≥ 18 y old elite- or sub-elite athletes from both sexes including para-athletes; *(iii)* experienced at least two consecutive weeks of lockdown, i.e., concomitant with the initial lockdown duration in many countries (between March – June 2020); *(iv)* had not missed training for greater than seven days due to illness/injury during the survey period; and *(v)* experienced medium-to-high lockdown severity (see below). The term “lockdown” is defined as *“large scale physical distancing measures and movement restrictions, to slow the COVID-19 transmission as a result of limited contact between people”* (www.who.int). In the context of our study, “lockdown-alone” (or LD) is referred to as lockdown *per se* or the period of lockdown without Ramadan fasting. A priori sample size estimation indicated that a minimum number of 5,484 participants were required (see Online Supplementary File 1). Informed consent was provided by participants under ethical approval in the spirit of the Declaration of Helsinki [22]. Data were collected and processed anonymously according to the guidelines of the General Data Protection Regulation (gdprinfo.eu, last visit: January 16^th^ 2022). Participation in the study was voluntary and all individuals were permitted to cease participation at any time before completing the survey.

A medium-to-high lockdown severity was met when one or more of the following criteria were fulfilled: *(i)* movement allowed for essential supplies and groceries only; *(ii)* access restrictions (i.e., closure, or limited access) to public exercise facilities (e.g., recreational areas such as parks or open spaces were prohibited and/or time/capacity limits imposed); and *(iii)* closure of an athlete’s training facilities at institutions, clubs, colleges, etc. [22].

### Study design

A cross-sectional, within-subject, descriptive study design was employed focusing on the distribution of frequencies and percentage of athletes in various demographic and/or comparative variables.

### Survey questionnaire

Survey questions were part of a wider international study examining the Effects of Confinement on Beliefs, Attitudes, and Training on Athletes (ECBATA consortium) [22]. The complete ECBATA survey can be found Open Access here [22]. In brief, the survey questions were developed by a core group of the research team, with face and construct validity verified by a second independent group of researchers, coaches and athletes. Test–retest reliability was determined within an English-speaking participant subgroup (n = 41), completing the survey twice approximately 9 days apart, with Cronbach’s alpha of > 0.81 (good reliability).

Questions assessed the athlete’s demographics, training knowledge, and attitudes/beliefs (i.e., termed “comparative variables”). Ramadan-specific questions from this original survey [which were not analysed in Washif et al. [22], given their focus on Muslim athletes that fasted] were utilised in the present study (see Table 1). These Ramadan specific questions explored changes in training load perception (primarily volume and intensity) and training time preference between RIFL and LD. The term “training load” is considered as a multidimensional construct that acts as a proxy measure to understand interactions between training/recovery induced adaptation and performance. In the current study, training load encompasses factors that affect training adaptation such as training volume and intensity, among others [23].

**TABLE 1:** Summary of comparative variables of athletes during COVID-19 lockdown including survey languages

	Category Comparative variables
1 Sex	Male, female
2 Age	*Grouped:* 18–29, 30–39, ≥40 years
3 Athlete classification	World class, international, national, state, recreational (or recreational-athlete)
4 Sport classification	*Classified:* Aquatic (e.g., surfing and swimming), combat (e.g., karate and silat), endurance (e.g., long-distance running, and triathlon), parasports (e.g., para-athletics and wheelchair tennis), power/technical (e.g., track and field, and weightlifting), precision (e.g., archery and lawn bowls), racquet (e.g., badminton and tennis), recreational (e.g., leisure and work-related), team (e.g., floorball and rugby), others (i.e., least known: aerial silks, etc.)
5 Country (current place or residence)	*Classified:* Africa, Asia, Europe, North America, Oceania, South America
6 Athlete status	Amateur, semi-professional, professional, others
7 Nine knowledge questions	*Summed-up and classified:* ≤ 50%: as poor, 51–70% as moderate, > 70% as good)
8 Seven belief/attitude questions	*Summed-up and classified:* ≤ 50% as poor, 51–70% as moderate, > 70% as good
9 Qualitative characterisation of overall training load, during Ramadan	*Grouped:* Reduced, maintained, increased
10 Qualitative characterisation of specific training load, during Ramadan	Decreased volume, decreased intensity, decreased volume and intensity, increased volume, increased intensity, increased volume and intensity
11 Qualitative characterisation of training time, during Ramadan	Afternoon, night, afternoon and night
12 Survey languages (total: 35)	English (*master version*), Albanian, Arabic, Bangla, Chinese-simplified, Chinese-traditional, Croatian, Czech, Danish, Finnish, French, German, Greek, Hindi, Indonesian, Italian, Japanese, Korean, Malay, Nepalese, Norwegian, Persian, Polish, Portuguese, Punjabi, Romanian, Russian, Sinhala, Slovenian, Spanish, Swahili, Swedish, Thai, Turkish, and Vietnamese

An online survey was administered and disseminated via Google Forms (17 May to 5 July 2020). The survey was shared via e-mail, messaging applications (e.g., WhatsApp^TM^, Signal^TM^, Telegram^TM^, etc.) and social media (e.g., Facebook^TM^, Twitter^TM^, and Instagram^TM^) through the professional networks of the research team (e.g., clubs, federations, and institutions). Using an English-language ‘master’ version, the survey was translated and administered in 34 other languages (see Table 1). Survey questions underwent translation and back-translation, performed by the research team (including at least one native speaker and one topic expert), including pilot completions of the survey and feedback from at least two native language speaking athletes, resulting in the finalised survey for all languages.

Data identified as duplicates, “incomplete” (i.e., where we deemed respondents clearly omitted answers), age-limit violations, and unmet lockdown severity were excluded (Figure 1). Data from questions with pre-set answers (i.e., pre-defined multiple choice) were converted directly into standardised codes/numbers, using an automated/customised setting on an Excel™ spreadsheet (Microsoft Corporation, Redmond, WA, USA). All automated responses were checked for veracity. The remaining data (i.e., free-text answers) underwent theme analysis/aggregation (all non-English responses were back-translated to English first), with subsequent themes classified into standardised codes/numbers to facilitate statistical analysis.

### Statistical Analysis

Statistical analyses were conducted using IBM SPSS Statistics for Windows, version 26.0 (IBM Corp., Armonk, NY, USA). Results are reported as frequencies and percentages for categorical variables. The variables were presented as mean ± standard deviation (SD). Relationships between the overall training load, overall training load during Ramadan, and specific training time preferences with categorical variables (demographics, sport classification, knowledge and beliefs) were assessed using a Chi-Square test for independence. Subsequently, analysis of adjusted residuals was performed to identify which subgroups (e.g., male vs female) contributed the most or the least to the relationships. Positive (i.e., higher) or negative (i.e., lower) residuals reflect the magnitude of the relationship(s). Any residual greater than 1.96 or less than -1.96 [24] was considered to be significant at p < 0.05. Sub-groups with extremely unequal and low frequencies can yield type 2 errors, and were therefore excluded or merged with other categories, where possible. Fisher’s exact test was also considered for the 2 × 2 Tables, when it was established that ‘variables had ≤ 20% of their expected count less than 5’ [25, 26]. A p-value of < 0.05 was considered significant.

## RESULTS

**FIG. 2:**
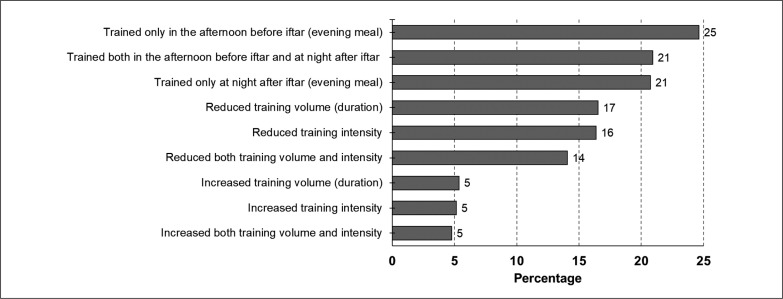
Overall training preference during Ramadan intermittent fasting with lockdown. **Question**: If you changed your training during the lockdown with Ramadan intermittent fasting, what did you do as compared to the lockdown without Ramadan?

All comparisons reflect changes from LD to RIFL. Overall preference in training changes: load (e.g., intensity and duration) and timing (e.g., before and after evening meal) are presented in Figure 2. A larger proportion of athletes (25%) preferred “training before the evening meal” with few athletes (5%) preferring to “increase training volume and intensity”.

Training load perceptions (i.e., decrease, maintain, increase) for comparative variables are presented in Table 2. During RIFL (relative to LD), more athletes decreased their training load (46–62%, dependant on comparative variables) than maintained (31–48%) or increased it (2–13%). Training load reductions [≥ 5% (p < 0.05)] were seen across several comparative variables, as follows: a greater reduction among athletes grouped in 30–39 than in 18–29 of ages; national > international athletes; team sports > precision sports; North America > European athletes; semi-professional > professional athletes; ‘good’ > ‘poor’ and ‘moderate’ beliefs/attitudes; ‘moderate’ > ‘poor’ knowledge.

**TABLE 2:** Overall training load (volume and intensity) during lockdown with Ramadan intermittent fasting compared to lockdown without Ramadan intermittent fasting.

	Decreased *row* (%)	Maintained *row* (%)	Increased *row* (%)	Total
**Sex**				
Male	58^a^	34^b^	8	3753
Female	53^b^	38^a^	9	1766
*Mean*	56	36	8	5519

**Age-group (years)**				
18–29	55^b^	36^a^	9	3905
30–39	60^a^	31^b^	9	1029
≥ 40	58	36	6	595
*Mean*	56	35	8	5529

**Athlete classification**				
World class	54	38	8	617
International	51^b^	41^a^	8	1171
National	59^a^	32^b^	8	2094
State	58	34	9	1324
Recreational-athlete	56	32	12^a^	322
*Mean*	56	35	8	5528

**Sport classification**				
Aquatic	55	35	10	251
Combat	59	36	6^b^	505
Endurance	53	37	10	805
Parasport	*	*	*	42
Power/technical	53	40^a^	8	543
Precision	46^b^	48^a^	6	156
Racquet	55	43^a^	2^b^	164
Recreational	52	35	13^a^	255
Team	59^a^	32^b^	9	2770
Other	*	*	*	38
*Mean*	56	35	8	5529

**Continents**				
Africa	60	31^b^	10	758
Asia	57	36	8	2717
Europe	53^b^	37	10	1455
North America	62^a^	30^b^	9	352
Oceania	*	*	*	15
South America	56	38	6	232
*Mean*	56	35	8	5529

**Athlete status**				
Amateur	56	35	9	2315
Semi-professional	60^a^	33^b^	8	1437
Professional	54^b^	38^a^	8	1731
Other	*	*	*	46
*Mean*	56	35	8	5529

**Knowledge**				
Poor	53^b^	36	10^a^	2169
Moderate	58^a^	35	7^b^	2407
Good	58	35	6^b^	953
*Mean*	56	35	8	5529

**Beliefs/attitudes**				
Poor	54^b^	36	10^a^	2471
Moderate	53^b^	39^a^	7	1247
Good	61^a^	32^b^	7^b^	1811
*Mean*	56	36	8	5529

Training load status in each category is % ‘yes’ answer relative to % ‘no’ answer;

a , significantly higher (in the same column);

b , significantly lower (in the same column);

* , excluded from assessment;

Specific changes in training load perceptions across the comparative variables are shown in Table 3. During RIFL, more athletes reduced either volume, intensity, or both volume and intensity (range: 7–21%, mostly 14–17%), than those who increased them (2–8%; mostly 5%). Reductions [≥ 5% (p < 0.05)] in training volume and intensity were seen across several comparative variables: national > world-class and state; combat > team sports; Africa > Europe and North America; Asia > North America; semi-professional > amateur athletes.

**TABLE 3:** Frequency and percentage of athletes that increased or decreased volume, intensity and both during the lockdown with Ramadan intermittent fasting compared to lockdown without Ramadan.

	↓ volume		↓ intensity		↓ volume & intensity		↑ volume		↑ intensity		↑ volume & intensity		Total
**Sex**	n	%	n	%	n	%	n	%	n	%	n	%	
Male	617	16	622	16	538	14	204	5	183	5	176	5	3753
Female	296	17	282	17	238	14	92	5	102	6	88	5	1766
*Total (Mean %)*	913	(17)	904	(16)	776	(14)	296	(5)	285	(5)	264	(5)	5519

**Age-group (years)**													
18–29	661	17	643	17	521	13^b^	223	6	225	6^a^	210	5^a^	3905
30–39	171	17	176	17	174	17^a^	52	5	45	4	36	4^b^	1029
≥ 40	82	14	86	15	82	14	21	4	15	3^b^	18	3	390
*Total (Mean %)*	914	(17)	905	(16)	777	(14)	296	(5)	285	(5)	264	(5)	5525

**Athlete classification**													
World class	95	15	100	16	70	11^b^	24	4	30	5	18	3^b^	617
International	198	17	190	16	165	14	73	6	72	6	61	5	1171
National	385	18^a^	375	18^a^	351	17^a^	96	4^b^	103	5	109	5	2094
State	188	14^b^	194	15	141	11^b^	83	6	67	5	65	5	1324
Recreational-nathlete	48	15	46	14	50	16	20	6	13	4	11	3	322
*Total (Mean %)*	914	(17)	905	(16)	777	(14)	296	(5)	285	(5)	264	(5)	5528

**Sport classification**													
Aquatic	46	18	33	13	38	15	15	6	19	8	13	5	251
Combat	95	19	103	20	94	19^a^	18	4	25	5	18	4	505
Endurance	135	17	119	15	116	14	40	5	41	5	37	5	805
Parasports	*		*		*		*		*		*		42
Power/technical	86	16	90	17	86	16	25	5	22	4	20	4	543
Precision	21	14	23	15	18	12	12	8	9	6	9	6	156
Racquet	30	18	31	19	30	18	10	6	9	6	4	2	164
Recreational	47	18	43	17	43	17	13	5	9	4	10	4	255
Team	444	16	450	16	343	12^b^	158	6	149	5	150	5	2770
Other	*		*		*		*		*		*		38
*Total (Mean %)*	914	(17)	905	(16)	777	(14)	296	(5)	285	(5)	264	(5)	5529

**Continents**													
Africa	159	21^a^	146	19^a^	143	19^a^	24	3^b^	31	4	24	3^b^	758
Asia	464	17	438	16	419	15^a^	148	5	141	5	127	5	2717
Europe	205	13^b^	252	17	163	11^b^	87	6	77	5	82	6	1455
North America	46	13	34	10^b^	25	7^b^	24	7	22	6	15	4	352
Oceania	*		*		*		*		*		*		15
South America	40	17	35	15	27	12	12	5	14	6	16	7	232
*Total (Mean %)*	914	(17)	905	(16)	777	(14)	296	(5)	285	(5)	264	(5)	5529

**Athlete status**													
Amateur	339	15^b^	323	14^b^	281	12^b^	128	6	108	5	101	4	2315
Semi-professional	270	19^a^	294	21^a^	240	17^a^	69	5	71	5	55	4^b^	1437
Professional	296	17	281	16	252	15	90	5	104	6	105	6^a^	1731
Other	*		*		*		*		*		*		46
*Total (Mean %)*	914	(17)	905	(16)	777	(14)	296	(5)	285	(5)	264	(5)	5529

**Knowledge**													
Poor	373	17	399	18^a^	334	15^a^	122	6	130	6^a^	88	4	2169
Moderate	363	15^b^	337	14^b^	303	13^b^	126	5	108	5^b^	120	5	2407
Good	178	19^a^	169	18	140	15	48	5	47	5	56	6	953
*Total (Mean %)*	914	(17)	905	(16)	777	(14)	296	(5)	285	(5)	264	(5)	5529

**Beliefs/attitudes**													
Poor	370	15^b^	443	18^a^	356	14	139	6	143	6	122	5	2471
Moderate	217	17	167	13^b^	168	14	61	5	63	5	48	4	1247
Good	327	18^a^	295	16	253	14	96	5	79	4	94	5	1811
*Total (Mean %)*	914	(17)	905	(16)	777	(14)	296	(5)	285	(5)	264	(5)	5529

% of yes answers;

a , significantly higher (in the same column);

b , significantly lower (in the same column);

* , excluded from assessment;

Changes in training time across comparative variables are detailed in Table 4. Athletes who altered lockdown training time during RIFL to perform training at both afternoon and night (13–29%), night only (12–26%), and afternoon only (18–36%) occurred disproportionally, depending on specific comparative variables. Changes [≥ 5% (p < 0.05)] in training time preferences were seen across the following variables; *(a) training both in afternoon and at night:* Athletes aged 18–29 y > 30–39 y and ≥ 40 y; combat > aquatic, endurance, and recreational; Asian > African and South American athletes; professional > amateur athletes; moderate > good knowledge; good > poor beliefs/attitudes; *(b) training at night only:* power/technical > combat and endurance; Asian > European and South American athletes; poor > moderate knowledge; poor > moderate knowledge; poor > moderate beliefs/attitudes; *(c) training in afternoon only*: national > world class and recreational-athlete; recreational > aquatic; African and North American > European athletes; semi-professional and professional > amateur athletes.

**TABLE 4:** Training time preferences during Ramadan with lockdown. **Question**: If you changed your training during the lockdown with Ramadan intermittent fasting, what did you do as compared to the lockdown without Ramadan?

	Within specific comparative variables, ‘yes’ answer						Total (n), ‘no’ + ‘yes’ answers
	Afternoon and Night		Night only		Afternoon only		
	n	%	n	%	n	%	n
**Sex**							
Male	788	21	800	21	904	24	3753
Female	368	21	346	20	450	26	1766
*Total (mean %)*	1156	(21)	1146	(21)	1354	(25)	5519

**Age-group (years)**							
18–29	893	23^a^	793	20	955	25	3905
30–39	181	18^b^	235	23	267	26	1029
> 40	83	14^b^	118	20	140	24	390
*Total (mean %)*	1157	(21)	1146	(21)	1362	(25)	5525

**Athlete classification**							
World class	117	19	122	20	132	21^b^	617
International	261	22	230	20	285	24	1171
National	436	21	420	20	564	27^a^	2094
State	294	22	291	22	315	24	1324
Recreational-athlete	49	15^b^	83	26^a^	66	21^b^	322
*Total (mean %)*	1157	(21)	1146	(21)	1362	(25)	5528

**Sport classification**							
Aquatic	40	16^b^	41	16	45	18^b^	251
Combat	145	29^a^	84	17^b^	136	27	505
Endurance	131	16^b^	139	17^b^	206	26	805
Parasports	*		*		*		42
Power/technical	107	20	138	25^a^	118	22	543
Precision	41	26	26	17	44	28	156
Racquet	44	27	31	19	41	25	164
Recreational	40	16^b^	62	24	88	35^a^	255
Team	591	21	603	22	669	24	2770
Other	*		*		*		38
*Total (mean %)*	1157	(21)	1146	(21)	1362	(25)	5529

**Continent**							
Africa	95	13^b^	157	21	275	36^a^	758
Asia	662	24^a^	638	24^a^	651	24	2717
Europe	295	20	244	17^b^	276	19^b^	1455
North America	71	20	79	22	106	30^a^	352
Oceania	*		*		*		15
South America	33	14^b^	27	12^b^	48	21	232
*Total (mean %)*	1157	(21)	1146	21	1362	25	5529

**Athlete status**							
Amateur	413	18^b^	472	20	482	21^b^	2315
Semi-professional	324	23	336	23^a^	388	27^a^	1437
Professional	409	24^a^	333	19	482	28^a^	1731
Other	*		*		*		46
*Total (mean %)*	1157	(21)	1146	(21)	1362	(25)	5529

**Knowledge**							
Poor	434	20	503	23^a^	544	25	2169
Moderate	552	23^a^	457	19^b^	593	25	2407
Good	171	18^b^	186	20	225	24	953
*Total (mean %)*	1157	(21)	1146	(21)	1362	(25)	5529

**Beliefs/attitudes**							
Poor	475	19^b^	593	24^a^	620	25	2471
Moderate	250	20	197	16^b^	315	25	1247
Good	432	24^a^	356	20	427	24	1811
*Total (mean %)*	1157	(21)	1146	(21)	1362	(25)	5529

*Afternoon + Night,* trained both in the afternoon before *iftar* (evening meal) and at night after *iftar; Night,* trained only at night after *iftar; Afternoon,* trained only in the afternoon before *iftar.* Note – may not add up to 100% due to non-compulsory question and multiple answer selection. Training time status in specific category is % ‘yes’ answer relative to % ‘no’ answer.

a , significantly higher (in the same column);

b , significantly lower (in the same column);

* , excluded from assessment;

## DISCUSSION

The main findings of the study indicated that RIFL compared to LD presented additional challenges for athletes during the first COVID-19 lockdown period. During RIFL, > 50% of athletes decreased their training loads independent of sex, age-group, athlete and sport classifications (excluding precision sports, 46%), continent, and training knowledge and beliefs/attitudes. Athletes reduced either training volume (~17%), intensity (~16%), or both (~14%) during RIFL compared to LD and they preferred to train at night (~21%) or in the afternoon (~25%), or twice a day [afternoon and night (~21%)]. For athletes who decided to alter training preferences during RIFL, their most preferred change was “training before *iftar*” (25%), and the least preferred change was “increase volume and intensity” (5%).

*Overall changes in training between RIFL and LD.* Research has shown that insufficient or sub-optimal caloric and fluid intake leading to reduced blood glucose levels and increased fatigue, will eventually compromise exercise performance in athletes who train while fasting [1, 6, 9]. The current findings showed that during RIFL, more athletes tended to reduce (46–62% dependant on comparative variables), rather than maintain (31–48%) or increase (2–13%) training loads, compared to LD. These perceptions were more apparent when comparisons were made for age-groups (younger or older athletes), athlete classification (Olympic through to lowest level) irrespective of geographical or national boundaries, athlete status (professional/amateur athletes), and those with different levels of training knowledge and beliefs/attitudes (Table 2). These changes, may in part be due to coach/athlete beliefs that training during Ramadan would be difficult to maintain [27]; and/or to a potential Ramadan nocebo effect [2]. Indeed, previous research has shown that during a soccer match, fasting players lowered playing intensity within the first 15 min of match-play, despite the absence of fatigue; which could be attributed to a feed-forward attempt to ration energy resources [9]. It appears that RIFL exacerbates the generally undesirable training alterations seen during Ramadan and LD, likely due to psycho-physiological effects which the present study was unable to delineate. It would be prudent for decision-makers responsible for RIFL athletes to develop educational materials and programs to support maintenance of minimum/optimal training to retain/progress athlete physical qualities including flexible training time/prescription, recovery promotion and the maintenance/support of athlete well-being.

*Specific changes in training frequency, volume, intensity between RIFL and LD.* During RIFL national-level (17%), combat sports (19%), African (19%) and Asian (15%) residents, and semi-professional (17%) athletes were more inclined to reduce both training volume and intensity compared to LD (Table 3). Reduced training loads during lockdown-associated challenges combined with RIF (i.e., RIFL) may have several explanations: increases in training load during a stressful period (i.e., lockdown) would have inevitably increased the physical demand (i.e., increased difficulty) during training [28]. As such, coaches would usually modify the training load due to the associated more challenging physiological and metabolic conditions when training during Ramadan [2]. Ideally, key training variables (e.g., volume and intensity) must be manipulated accordingly to elicit specific adaptive responses [29]. Furthermore, mobility restrictions and limited food choices during lockdown [14, 20], could decrease the daily energy intake among athletes, a situation that could be exacerbated during RIF. Such reductions may be partially explained by the fact that the same exercise implemented in a fasted state increases perceived exertion and difficulty [30], prompting athletes to lower their training loads. Thus, it is possible that the training loads could be influenced by the athletes themselves, and how they coped/managed the given training intensity and volume.

RIF may increase feelings of lethargy, low motivation, less enjoyment in exercise or training – compounded by lockdown. Indeed, social interactions with other familiar (i.e., teammates) and non-familiar athletes could provide some form of “external” motivation to work and exercise harder during the sessions [31]. It may be argued that one potential issue with training/exercising in the RIFL period is exacerbation of low-morale and self-esteem of athletes to perform training. It is known that excessive stress due to training and non-training (e.g., lockdown-related turbulence) may predispose an athlete to overtraining, increased injury risk, or acute illness [32]. In this sense, our findings reflect what the athletes/coaches perceived or were able to perform when training under RIFL (i.e., mostly reduced training loads). Interestingly, an earlier study [33] reported that the negative effects of RIF on some athletes were not observed in elite judoists who maintained the same training loads during Ramadan to those seen pre-Ramadan. Usually, such statements hold true for those who consume appropriate meals (sufficient calories), hydrate adequately during the night non-fasting period, and maintain good sleep throughout the month of Ramadan [34].

*Changes in training time preference between RIFL and LD.* In the present study, we identified that a greater proportion of athletes reported training one single session, i.e., only in the afternoon (18–36%) more than only at night (12–26%) or twice a day (i.e., afternoon and night: 13–29%). It appears there is no exclusive training time that was more preferred than the others in the current study, and that was dependent on specific sub-categories (Table 4). One possible explanation for this outcome is that, while in lockdown, athletes did not need to travel to training grounds and competitions prompting them to choose their own preferred ‘’home’’ training time. Nevertheless, training close to sunset can benefit from post-training food intake before the next dawn meal. Such a strategy may promote adaptations to the exercise performed [35], although it occurs long after the last nutrient intake (*sahour*). Moreover, training at night may be convenient but it can alter sleep patterns [5, 35]. Indeed, training efforts at night can delay bedtime by three hours, although partially compensated by two hours additional sleep during the day [36].

In summary, during RIFL, a small number of athletes decided to increase training load, which is reasonable given that any increases during a stressful period of lockdown would have increased the overall physical demand (i.e., increased difficulty) of the training itself [28]. While changes in training were up to 25% for different training load and preference (Figure 2), it cannot be disregarded that some athletes maintained their lockdown training behavior during RIFL. Training during RIFL might have exposed “health issues” such as fatigue, dizziness, sleep deprivation, irritability, and headaches. Thus, it is important to adhere to healthy practices, including sleep hygiene, appropriate hydration during non-fasting period, and other lifestyle recommendations [37].

*Methodological considerations.* Some limitations of the study need to be acknowledged. First, the use of external subjective measures (self-assessment questionnaire) to report information related to training loads could obviously be subject to misreporting. Objective measures (e.g., physiological responses using a heart rate monitor) would be ideal, but not easily obtainable in such a study setting. Thus, we used a self-reported online survey to access a large number of athletes i.e., from > 100 countries and six continents. Secondly, we acknowledge the reported changes in training loads during Ramadan were primarily based on experience, self-preference, and beliefs of the athletes. Nevertheless, we used simple closed questions to facilitate the athlete’s responses to limit the degree of misinterpretation. Thirdly, the frequency or size of our sample was disproportionally distributed between the sub-groups or comparative variables (e.g., low representation of Oceania and parasports). These sub-groups were merged where possible (e.g., age-group), or otherwise excluded from the statistical analyses. Fourthly, it is possible that non-Muslim or non-fasting Muslim athletes filled out the Ramadan survey questionnaire, or athletes who mistakenly or deliberately mis-claimed they belonged to certain classification (e.g., worldclass), which could limit the study’s conclusions to some extent. Although such actions are beyond our control, all responses were checked for veracity, including data consistency and click-through behaviours. The large study sample likely limits the influence of such errors on the overall results. Fifthly, the lack of some key metrics known to influence athletes’ practice/choice, such as daily fasting duration, Ramadan season (ambient temperature and humidity), number of years of experience of the athletes in terms of training while fasting during Ramadan, were not recorded. However, the study’s conclusions were based on the general results of a highly heterogenous sample (in age, sports, lockdown severity, etc.), and likely represent the athlete’s real-life practices. Finally, the results of the present study concern the early phase of the COVID-19 pandemic (2020), and therefore their extrapolation to the successive Ramadan months (i.e., 2021 and beyond) should be considered with caution. Notwithstanding these limitations, we analysed training load changes and time preference in a large number of athletes worldwide, which improved the reliability of the study [38], uniquely represent a large population of athletes and sports, and likely reflect the reality the athletes have experienced through during RIFL.

## CONCLUSIONS

There were clear alterations in training loads during RIF while athletes were in lockdown (i.e., RIFL) relative to lockdown-only (i.e., LD). More athletes reduced rather than maintained or increased their training load evidenced by reduced training volume, intensity, or both. This outcome indicates that athletes perceived training during Ramadan to be even more challenging than during lockdown. Overall, the influence of specific categories (e.g., sex, age-groups, athlete and sport classifications) were generally < 10%, but should not be underestimated. It also appears that changes in training time due to RIFL seem centred before *iftar*, although some athletes reported training after *iftar* or both (before and after), which may be based on the athlete’s preference. Future studies should investigate athlete’s perceptions and/or physiological responses associated with lockdown-related situations for managing training and competitive performance before-, during-, and after-lockdown with concurrent RIF.

## Practical Applications

– Training loads of athletes were reduced from lockdown-only to lockdown with Ramadan intermittent fasting, indicating necessary adjustments and/or possibly additional challenges experienced by athletes.
– For changes in training loads, the influence of specific categories (e.g., sex, age-groups, athlete, and sport classifications) varied, and therefore, *(i)* certain training and educational supports could potentially be given similarly for all fasting athletes during lockdown; *(ii)* while also cognisant of athletes who are more vulnerable for implementation of athlete-specific support.
– When lockdown and Ramadan occurs concurrently, flexible training times may be preferred by athletes (usually, immediately before *iftar*) to accommodate daily training requirements and the challenges they encounter.

## SUPPLEMENTARY FILE 1

The sample size was calculated [1] as follows 1: N = [(Z_α/2_)^2^ × p × (1 – p) × D]/E^2^, where

*N*, was the number of needed Muslim athletes;

*Zα/2*, normal deviate for two-tailed alternative hypothesis at a level of significance;

*E*, margin of error;

*D*, design (= 1 for simple random sampling); and

*p*, proportion of the main event of interest [i.e., frequency of Muslim athletes who decreased their training load during RIFL (compared to Lockdown-only)].

Given the pioneering character of our study, “p” was taken from a previous study [2] aiming to evaluate whether COVID-19 restrictions and RIF are associated with poor physical activity. The authors noted that 33.7% (p = 0.337) of the total sample (n = 510 adults from the general population) reported less physical activity during Ramadan. Assuming a confidence interval of 99% (*Zα/2* = 3.29) and an “*E*” of 2.1%, the total sample size was 5,484 athletes (5,484 = 3.292 0.337 (1–0.337)/0.0212)
